# Supplementary material for: OTULIN maintains skin homeostasis by controlling keratinocyte death and stem cell identity
Source: Nat Commun. 2021 Oct 8;12:5913. doi: 10.1038/s41467-021-25944-2 (PMC8501048; doi:10.1038/s41467-021-25944-2)
Supplement: Supplementary file 1 — Supplementary Information [file 41467_2021_25944_MOESM1_ESM.pdf]

Supplementary Information for:

OTULIN maintains skin homeostasis by controlling keratinocyte death and stem cell identity

Esther Hoste, Kim Lecomte and Geert van Loo

Supplementary Figures 1-7

Supplementary Table 1 and 2

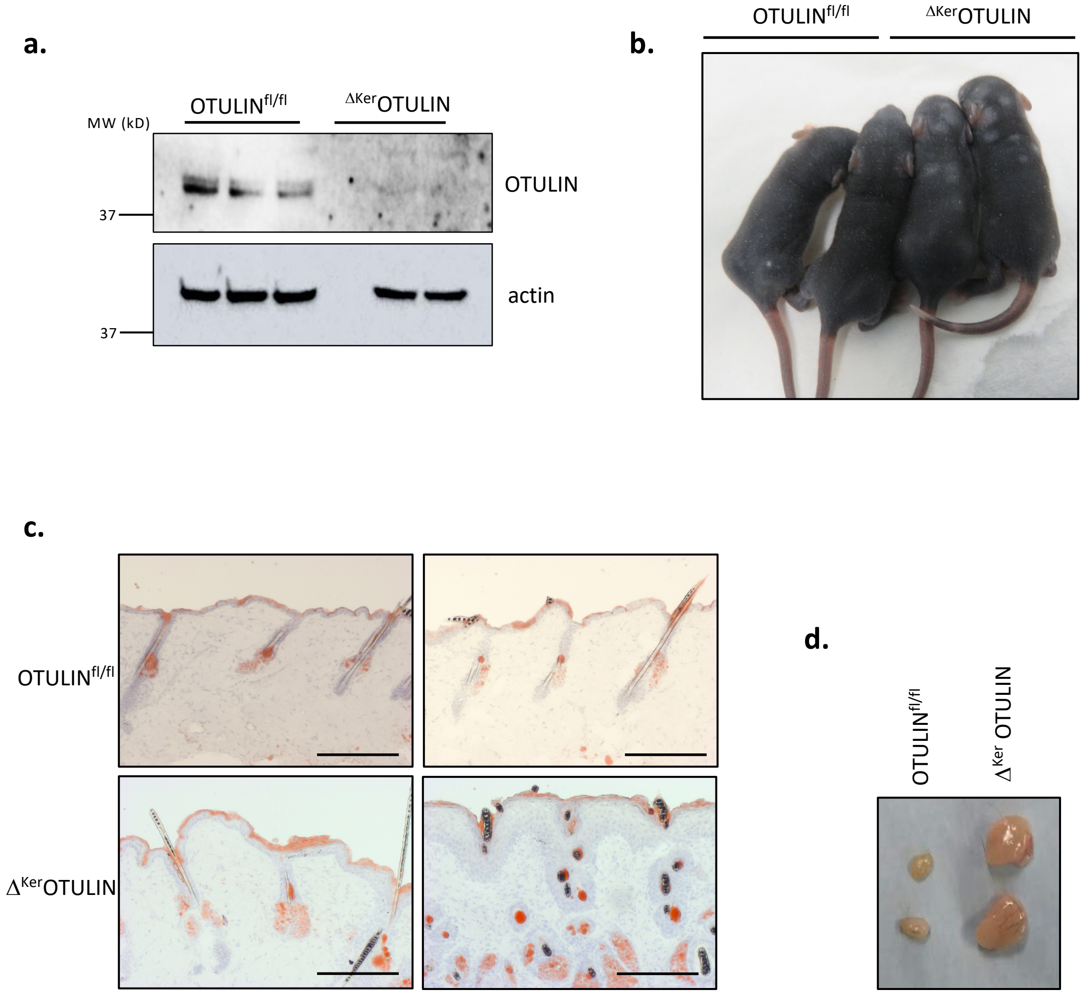

Supplementary Figure 1. a. Western blot analysis of OTULIN on lysates from PMK cultures isolated from OTULIN$^{fl/fl}$ and Δ^{Ker}OTULIN mice **b.** Representative picture of P6 OTULIN$^{fl/fl}$ and Δ^{Ker}OTULIN mice. **c.** Oil Red O staining of skin sections obtained from back skin of 7-weeks old OTULIN$^{fl/fl}$ and Δ^{Ker}OTULIN mice. Scale bars: 100 μm. **d.** Representative photograph of inguinal lymph nodes of 7-weeks old mice of the indicated genotypes.

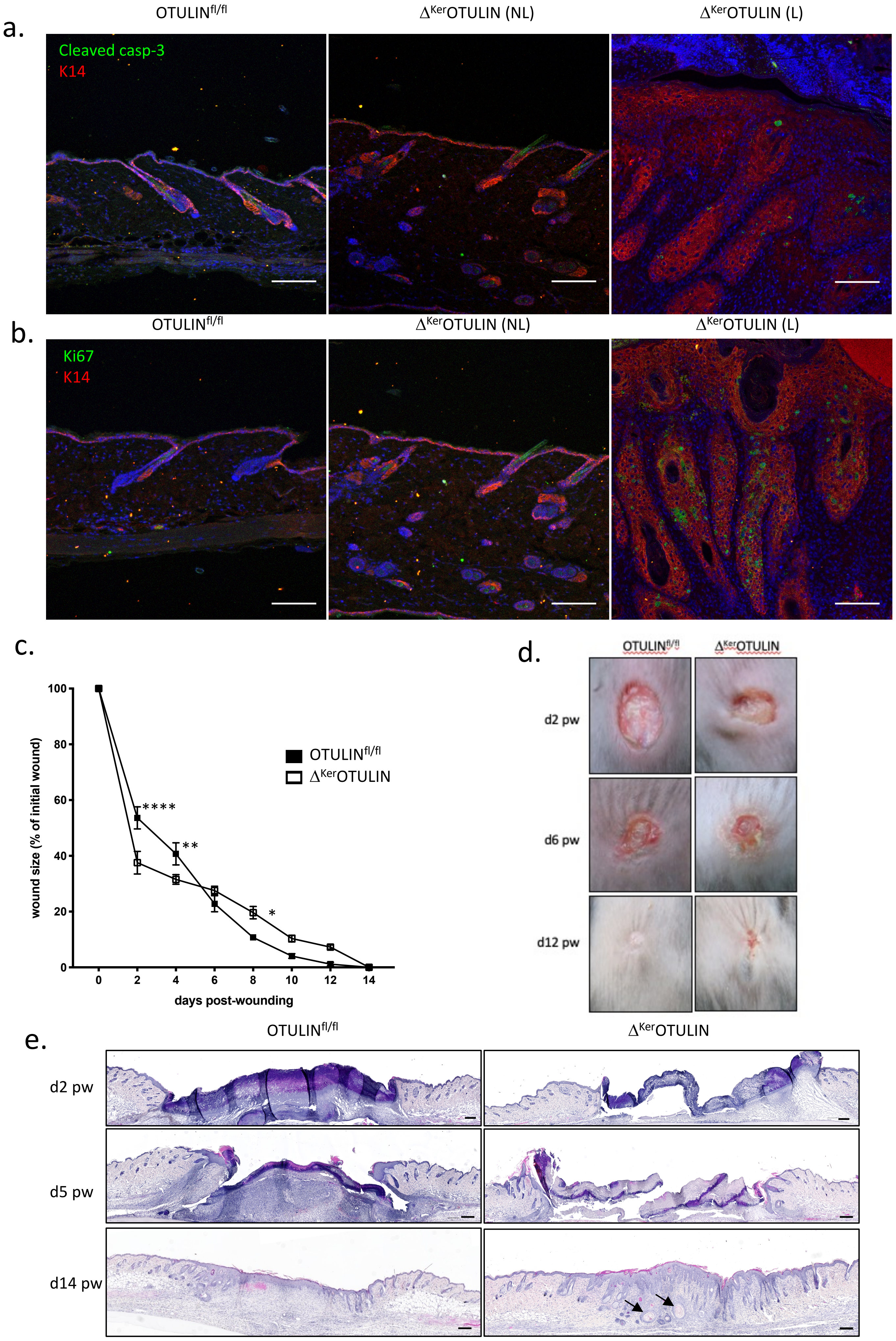

Supplementary Figure 2. a. Immunofluorescent staining of skin sections from 7-weeks old OTULIN$^{fl/fl}$ mice and non-lesional (NL) and lesional (L) skin of Δ^{Ker}OTULIN mice with antibodies against cleaved caspase-3 (green), keratin-14 (red) and nuclear staining with Dapi. Scale bar: 100 μm. **b.** Immunofluorescent staining of skin sections from 7-weeks old OTULIN$^{fl/fl}$ mice and non-lesional (NL) and lesional (L) skin of Δ^{Ker}OTULIN mice with antibodies against Ki-67 (green), keratin-14 (red) and nuclear staining with Dapi. Scale bar: 100 μm. **c.** Wound healing dynamics in OTULIN$^{fl/fl}$ (n=8 mice) and Δ^{Ker}OTULIN (n=6 mice) mice that were wounded at 7-weeks old (* p=0.036; ** p=0.00284; **** p<0.0001; Two-way ANOVA with multiple comparisons). Data represent means ± SEM. Scale bar: 200 μm. **d.** Representative photographs of wounds of OTULIN$^{fl/fl}$ and Δ^{Ker}OTULIN mice at the indicated time-points post wounding (pw). **e.** H&E-stained sections of wounds OTULIN$^{fl/fl}$ and Δ^{Ker}OTULIN mice at the indicated days pw. Scale bars: 200 μm.

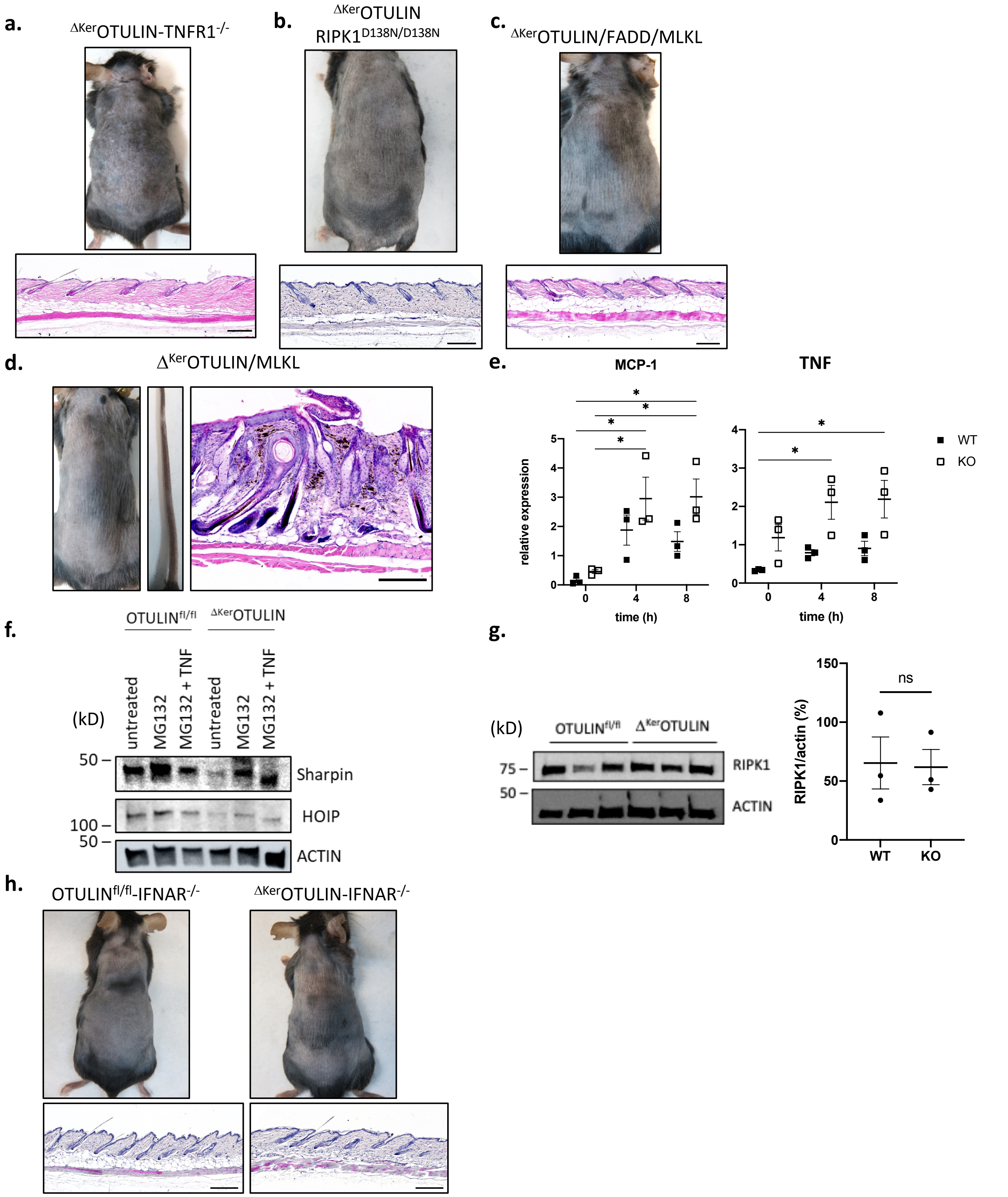

Supplementary Figure 3. a. Representative images and H&E-stained skin sections of 42-weeks old Δ^{Ker}OTULIN-TNFR1$^{-/-}$ mice. **b.** Representative images and H&E-stained skin sections of 42-weeks old Δ^{Ker}OTULIN-RIPK1$^{D138N/D138N}$ mice. **c.** Representative images and H&E-stained skin sections of 40-weeks old Δ^{Ker}OTULIN/FADD/MLKL mice. **d.** Representative images and H&E- stained skin sections of 8-weeks old Δ^{Ker}OTULIN/MLKL mice. Scale bars for panel a to d: 200 μm. **e.** Relative mRNA expression of MCP-1 and TNF in lysates of OTULIN$^{fl/fl}$ (WT; n=3) and Δ^{Ker}OTULIN (KO; n=3) PMKs. Data represent means ± SEM. (* $p < 0.05$; Two-way ANOVA with multiple comparisons). **f.** Western blot analysis on lysates from PMK cultures isolated from OTULIN$^{fl/fl}$ and Δ^{Ker}OTULIN mice that were treated with or without TNF in the presence of the proteasome inhibitor MG132 (20 mM). **g.** Immunoblotting for RIPK1 in epidermal tail lysates of OTULIN$^{fl/fl}$ and Δ^{Ker}OTULIN mice and associated densitometry analysis relative to ß-actin levels. **h.** Representative images and H&E-stained skin sections of 40-weeks old OTULIN$^{fl/fl}$ IFNAR$^{-/-}$ and Δ^{Ker}OTULIN IFNAR$^{-/-}$ mice. Scale bar: 200 μm.

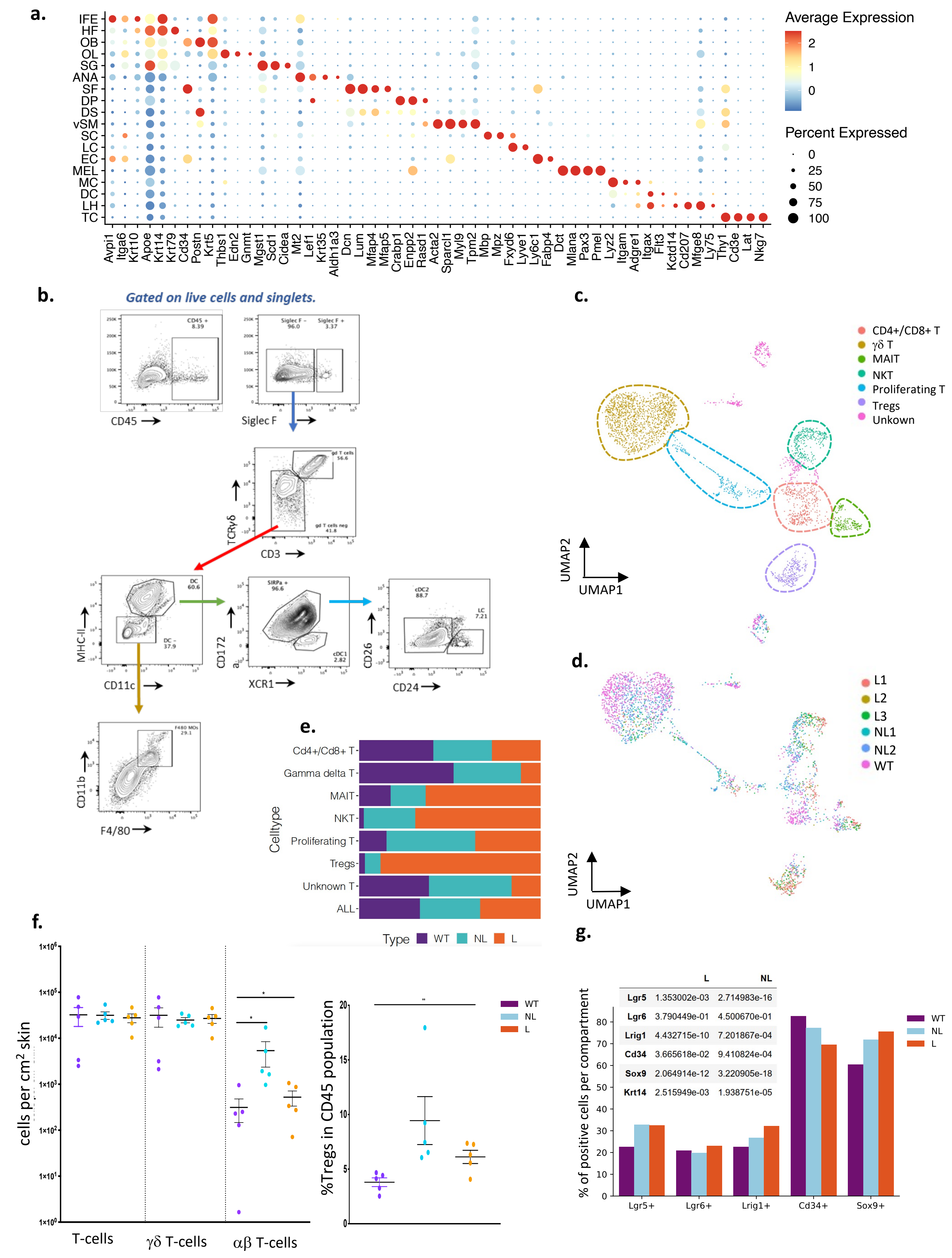

Supplementary Figure 4. a. Dot plot showing expression of selected differentially expressed genes per annotated cluster as adapted from Joost et al. [35]. The dot size represents the percentage of cells expressing the gene, and the color represents the average expression of that gene within a cluster. **b.** Gating strategy for flow cytometric analysis of immune cell-types infiltrating the skin. **c.** UMAP plot of annotated

T-cell subcluster, showing several assigned clusters by unbiased clustering. Tregs segregate based on differential *Foxp3*, *CD4* and *IL10* gene expression. CD8 T-cells segregate in a cluster together with CD4 T-cells identified by *CD8* or *CD4* expression, $\gamma\delta$ T-cells were identified by lack of *CD8* and *CD4* expression. **d.** Distribution of WT, NL and L cells within the T-cell subclusters. **e.** Relative normalized T-cell frequencies of the indicated populations in the different conditions. **f.** Flow cytometric analysis of T-cell composition in control OTULIN$^{fl/fl}$ skin (WT, n=5 mice) and non-lesional (NL) and lesional (L) skin of Δ^{Ker}OTULIN mice (n=5 mice per condition). The absolute number of T-cells per cm^2 skin is plotted. Right panel depicts the percentage of FoxP3-positive Tregs within the CD45 population, data represent means $\pm$ SEM (* $p<0.05$; ** $p=0.0079$; Mann-Whitney two-sided testing). **g.** Percentage of cells positive for the indicated markers present in the permanent part of the epidermis (incl. IFE, SG, uHF and HF bu) and associated p-value (Chi-squared test, comparing L or NL gene frequencies to expected frequencies from WT).

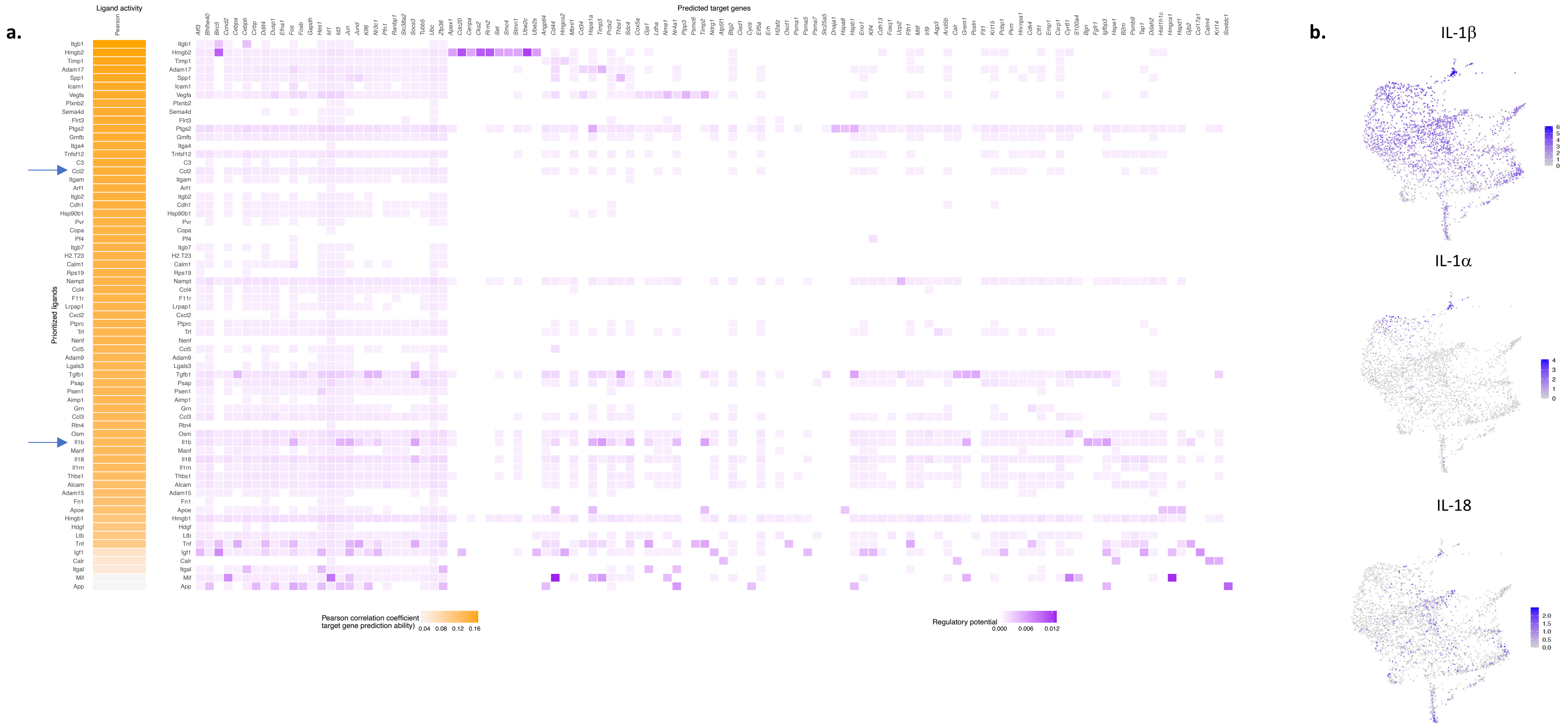

Supplementary Figure 5. a. Schematic representation of NicheNet analysis identifying ligands secreted by innate immune cells that bind on receptors in keratinocytes, mediating gene expression changes. Non-lesional Δ^Ker^OTULIN skin is compared to WT skin. CCL2 (MCP-1) and IL-1b are indicated with an arrow. **b.** Feature plot of IL-1β, IL-1α and IL-18 mRNA expression levels in innate immune cells, as determined by scRNAseq.

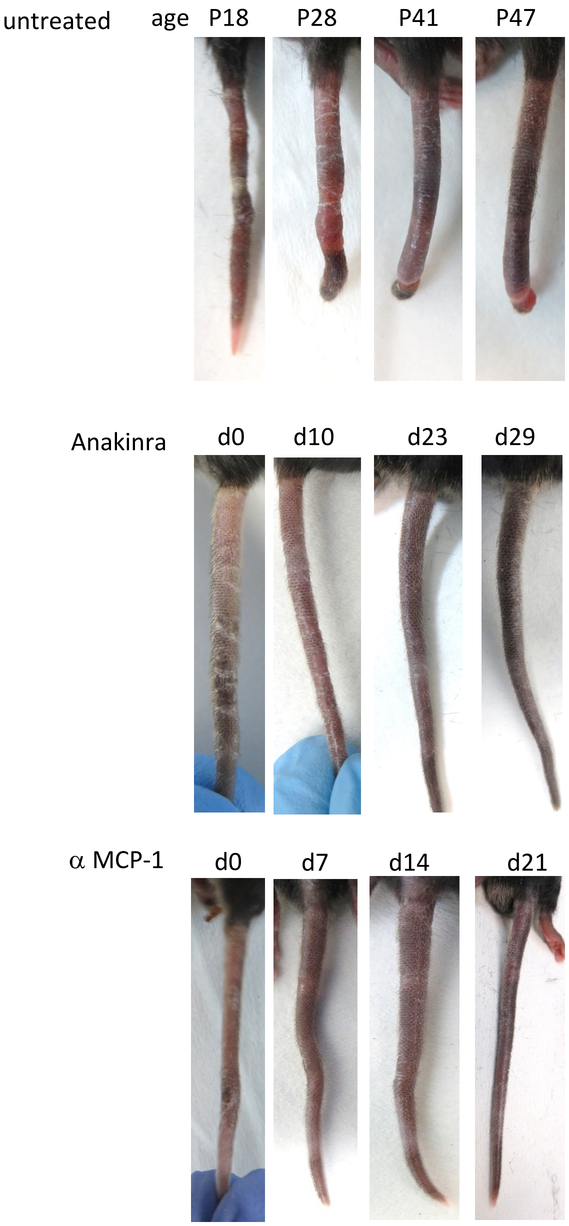

Supplementary Figure 6. Representative pictures of tail of Δ^{ker} OTULIN mice treated with daily injections of 300 mg/kg Anakinra or 40 mg/kg a-MCP-1 antibody twice weekly, and untreated controls. Duration of treatment is indicated.

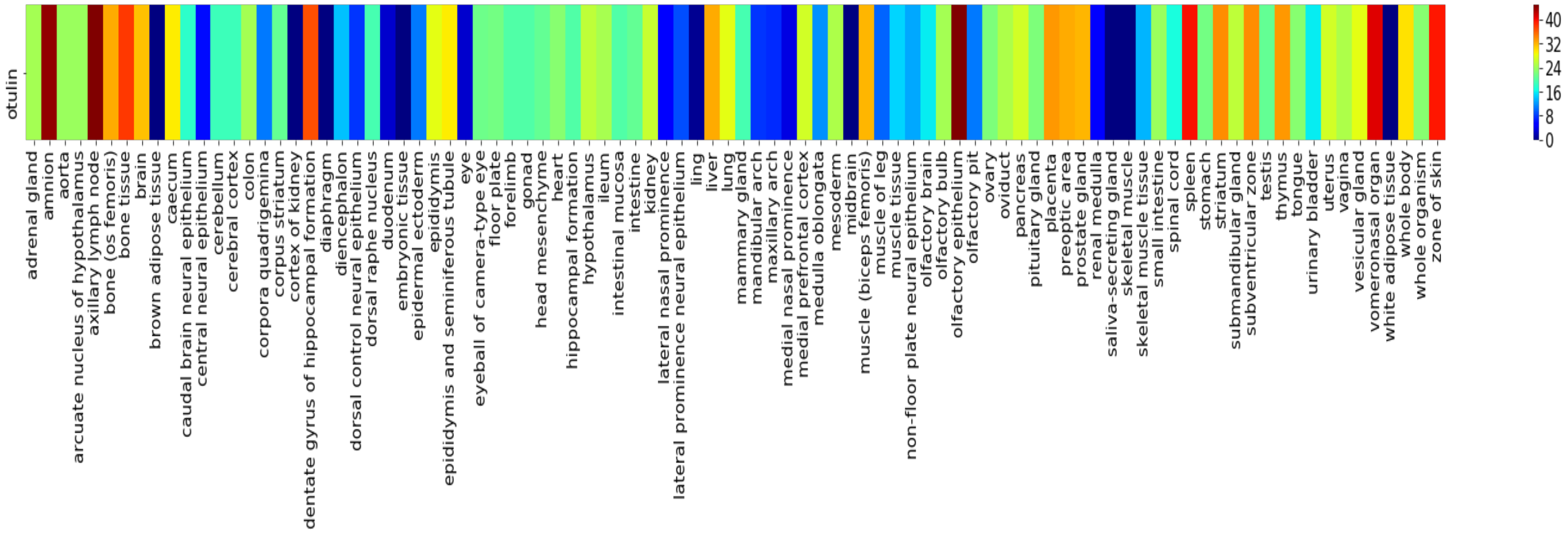

Supplementary Figure 7. Average *OTULIN* mRNA expression levels in different tissues of the mouse, as assessed by the EBI tissue expression atlas.

Antibodies	Cat #	company	dilution	application
Anti-MCP-1	BE0185	InVivoMab	40 mg/kg	Therapeutic treatment
CD24-eFluor450	48024282 (clone M1/69)	eBioscience	1/200	Flow cytometry
CD3-PacBlue	100214 (clone 17A2)	Biolegend	1/100	Flow cytometry
CD127a-PerCP-eFluor710	46172182 (clone P84)	eBioscience	1/200	Flow cytometry
CD11b-BV605	563015 (clone M1/70)	BD Bioscience	1/200	Flow cytometry
cD11c-BV711	cD11c-BV711	BD Pharmingen	1/200	Flow cytometry
F4/80-BV785	123141 (clone BM8)	Biolegend	1/400	Flow cytometry
CD45-AF700	56045182 (clone 30-F11)	eBioscience	1/400	Flow cytometry
MHCII-APC-eFluor780	47532182 (clone M5/114152)	eBioscience	1/400	Flow cytometry
Siglec-F-PE	552126 (clone E502440)	BD Pharmingen	1/1000	Flow cytometry
B220-PE-Cy5	553091 (clone RA36B2)	BD Pharmingen	1/500	Flow cytometry
CD317-PE-Cy7	25317282 (clone eBio927)	eBioscience	1/200	Flow cytometry
CD3-BUV395	563565 (clone 1452C11)	BD Bioscience	1/100	Flow cytometry
TCRb chain PE/Cy7	109222 (clone H57597)	Biolegend	1/300	Flow cytometry
FoxP3-APC	17577382	eBioscience	1/100	Flow cytometry
TCRγδ-AF488	118128 (clone GL3)	Biolegend	1/200	Flow cytometry
Fc block CD16/CD32	553142 (clone 2.4G2)	BD Bioscience	1/400	Flow cytometry
Filaggrin	PRB-417P	Covance	1/1000	immunofluorescence
F4/80	MCA497G	AbD serotec	1/1000	immunofluorescence
CD11b-PE	12-0112-81 (clone M1/70)	eBioscience	1/300	immunofluorescence
CD45	550539 (clone 30-F11)	BD Pharmingen	1/500	immunofluorescence
Keratin-6A	PRB-169P	Biolegend	1/1000	immunofluorescence
Keratin-14	Ab7800 (clone LL002)	Abcam	1/1000	immunofluorescence
Ki-67	12202 (clone D3B5)	Cell Signalling Technology	1/1000	immunofluorescence
Cleaved casp-3	9661	Cell Signalling Technology	1/1000	immunofluorescence
Donkey-anti-mouse AF488	A-21202	Thermofisher	1/2000	immunofluorescence
Goat-anti-rabbit DyLight 555	84541	Thermofisher	1/2000	immunofluorescence
Donkey-anti-rabbit AF488	A21206	Thermofisher	1/2000	immunofluorescence
Goat-anti-mouse DyLight 555	84540	Thermofisher	1/2000	immunofluorescence

OTULIN	14127	Cell Signalling Technology	1/1000	Western blotting
JNK	554285	BD Bioscience	1/1000	Western blotting
phospho-JNK	PS1019	Millipore	1/1000	Western blotting
IκBα	sc371	Santa Cruz Biotechnology	1/1000	Western blotting
phospho-IκBα	9246	Cell Signalling Technology	1/1000	Western blotting
p38 MAPK	CST9212	Cell Signalling Technology	1/1000	Western blotting
phospho-p38 MAPK	CST9215	Cell Signalling Technology	1/1000	Western blotting
HOIL-1		kind gift of Dr. Henning Walczak	1/2000	Western blotting
HOIP		kind gift of Dr. Rune Damgaard	1/1000	Western blotting
SHARPIN	14626-1-AP	Proteintech	1/1000	Western blotting
RIPK1	3493	Cell Signalling Technology	1/2000	Western blotting
linear ubiquitin	MABS451 (LUB9)	Millipore	1/2500	Western blotting
caspase-3	9662	Cell Signalling Technology	1/1000	Western blotting
actin-HRP	sc-47778	Santa Cruz Biotechnology	1/10000	Western blotting
anti-rabbit HRP	NA931	Amersham	1/2500	Western blotting
anti-mouse HRP	NA934	Amersham	1/2500	Western blotting
anti-goat HRP	Sc-2354	Amersham	1/2500	Western blotting

Supplementary Table 1. List of used antibodies

Gene	Forward primer	Reverse primer
Cxcl10	CCAAGTGCTGCCGTCATTTTC	GGCTCGCAGGGATGATTTCAA
GAPDH	TGAAGCAGGCATCTGAGGG	CGAAGGTGGAAGAGTGGGAG
Ifitm3	TGTTACACCTGCGTGTAGGG	GATGGTGGGTGATGTGACTG
Ifna2	GCCATCCCTGTGCTGCGAGA	GCAGCAGATGAAGCCTTTGATGTGA
Ifnb1	AGCTCCAAGAAAGGACGAACA	GCCCTGTAGGTGAGGTTGAT
IL13	TGTGTAGCTGAGCAGTTTTGT	TTCTGTGTAGCCCTGGATTCC
IL4	TGGACTCATTCATGGTGCAG	AACATGGGAAAACTCCATGC
IL6	GAGGATACCACTCCCAACAGACC	AAGTGCATCATCGTTGTTCATACA
Isg15	ACGGTCTTACCCTTTCCAGTC	CCCCTTTCGTTCCTCACCAG
MCP-1	TTAAAAACCTGGATCGGAACCAA	GCATTAGCTTCAGATTTACGGGT
Mx1	GACCATAGGGGTCTTGACCAA	AGACTTGCTCTTTCTGAAAAGCC
S100A8	ATCCTTTGTCAGCTCCGTCTT	TGTAGAGGGCATGGTGATTTC
ß-actin	GCTTCTAGGCGGACTGTTACTGA	GCCATGCCAATGTTGTCTCTTAT
TNF	ACCCTGGTATGAGCCCATATAC	ACACCCATTCCCTTCACAGAG

Supplementary Table 2: List of used primers
