# Supplementary material for: OTULIN maintains skin homeostasis by controlling keratinocyte death and stem cell identity
Source: Nat Commun. 2021 Oct 8;12:5913. doi: 10.1038/s41467-021-25944-2 (PMC8501048; doi:10.1038/s41467-021-25944-2)
Supplement: Supplementary file 3 — Reporting Summary [file 41467_2021_25944_MOESM3_ESM.pdf]

nature portfolio

Corresponding author(s): Esther Hoste and Geert van Loo

Last updated by author(s): 2021/08/16

Reporting Summary

Nature Portfolio wishes to improve the reproducibility of the work that we publish. This form provides structure for consistency and transparency in reporting. For further information on Nature Portfolio policies, see our Editorial Policies and the Editorial Policy Checklist.

Statistics

For all statistical analyses, confirm that the following items are present in the figure legend, table legend, main text, or Methods section.

n/a	Confirmed	
☐	☒	The exact sample size (*n*) for each experimental group/condition, given as a discrete number and unit of measurement
☐	☒	A statement on whether measurements were taken from distinct samples or whether the same sample was measured repeatedly
☐	☒	The statistical test(s) used AND whether they are one- or two-sided<br>*Only common tests should be described solely by name; describe more complex techniques in the Methods section.*
☐	☒	A description of all covariates tested
☐	☒	A description of any assumptions or corrections, such as tests of normality and adjustment for multiple comparisons
☐	☒	A full description of the statistical parameters including central tendency (e.g. means) or other basic estimates (e.g. regression coefficient) AND variation (e.g. standard deviation) or associated estimates of uncertainty (e.g. confidence intervals)
☐	☒	For null hypothesis testing, the test statistic (e.g. *F*, *t*, *r*) with confidence intervals, effect sizes, degrees of freedom and *P* value noted<br>*Give P values as exact values whenever suitable.*
☒	☐	For Bayesian analysis, information on the choice of priors and Markov chain Monte Carlo settings
☒	☐	For hierarchical and complex designs, identification of the appropriate level for tests and full reporting of outcomes
☐	☒	Estimates of effect sizes (e.g. Cohen's *d*, Pearson's *r*), indicating how they were calculated

Our web collection on statistics for biologists contains articles on many of the points above.

Software and code

Policy information about availability of computer code

Data collection	Demultiplexing of the raw data and mapping to the mouse genome mm10 (1.2.0) was done by the 10X Cell Ranger software (version 2.1.1; cellranger). Preprocessing of the data was done by the scran and scater R package (version 4.1) according to workflow proposed by the Marioni and Theis lab.
Data analysis	For single-cell RNA-sequencing analysis: A principal component analysis (PCA) plot was generated as an extra filtering using the run PCA function from the scater R package with the default parameters. Genes expressed in less than 3 cells and cells expressing less than 200 genes were removed. The samples were aggregated using the merge function, counts were normalized and log2 transformed using the NormalizeData function, both from the Seurat R package (v3.1.0) using default parameters. Detecting highly variable genes, finding clusters and creating UMAP plots was done using the Seurat pipeline. Clustering was performed using the first 34 principal components and a resolution of 0.8.<br>An open-source R implementation of NicheNet analysis software is available at GitHub (https://www.github.com/saeyslab/nichenetr).<br>Prism GraphPad 9 software was used to perform ANOVA and T-testing.<br>For flow cytometry analysis, we made use of FLowJo version 10.6.1.

For manuscripts utilizing custom algorithms or software that are central to the research but not yet described in published literature, software must be made available to editors and reviewers. We strongly encourage code deposition in a community repository (e.g. GitHub). See the Nature Portfolio guidelines for submitting code & software for further information.

Data

Policy information about availability of data

All manuscripts must include a data availability statement. This statement should provide the following information, where applicable:

- Accession codes, unique identifiers, or web links for publicly available datasets
- A description of any restrictions on data availability
- For clinical datasets or third party data, please ensure that the statement adheres to our policy

The accession number for the raw scRNA-sequencing data reported in this study is Gene Expression Omnibus (GeO): GSE162394 (https://www.ncbi.nlm.nih.gov/geo/query/acc.cgi?acc=GSE162394).

Field-specific reporting

Please select the one below that is the best fit for your research. If you are not sure, read the appropriate sections before making your selection.

- [x] Life sciences
- [ ] Behavioural & social sciences
- [ ] Ecological, evolutionary & environmental sciences

For a reference copy of the document with all sections, see nature.com/documents/nr-reporting-summary-flat.pdf

Life sciences study design

All studies must disclose on these points even when the disclosure is negative.

Sample size	Preliminary experiments were performed when possible to determine sample size, taking into account resources available and ethical, reductionist animal use.
Data exclusions	No samples or data were excluded from analyses.
Replication	Experiments were replicated as indicated in the appropriate figure legends. All attempts of replication were successful.
Randomization	All animals were assigned to groups based on their genotype
Blinding	As the phenotype of keratinocyte-specific OTULIN-deficient mice is macroscopically apparent, analysis could not be performed in a blinded fashion. For in vitro experiments: all analyses were performed in a blinded fashion.

Reporting for specific materials, systems and methods

We require information from authors about some types of materials, experimental systems and methods used in many studies. Here, indicate whether each material, system or method listed is relevant to your study. If you are not sure if a list item applies to your research, read the appropriate section before selecting a response.

Materials & experimental systems

n/a	Involved in the study	
[ ]	[x]	Antibodies
[x]	[ ]	Eukaryotic cell lines
[x]	[ ]	Palaeontology and archaeology
[ ]	[x]	Animals and other organisms
[x]	[ ]	Human research participants
[x]	[ ]	Clinical data
[x]	[ ]	Dual use research of concern

Methods

n/a	Involved in the study	
[x]	[ ]	ChIP-seq
[ ]	[x]	Flow cytometry
[x]	[ ]	MRI-based neuroimaging

Antibodies

Antibodies used	Commercial antibodies were used. All antibodies used are listed in Supplementary Table 1. Cells were stained with the following fluorochrome-linked antibodies for flow cytometry: CD24-<br>eFluor450 (eBioscience, M1/69, 48024282), CD3-PacBlue (Biolegend, 17A2, 100214), L/D Amcyan (Thermofisher, L34957), CD127a-PerCP-eFluor710 (eBioscience, P84, 46172182), CD11b-BV605 (BD Bioscience, M1/70, 563015), cD11c-BV711 (BD Pharmingen, HL3, 563048), F4/80-BV785 (Biolegend, BM8, 123141), CD45-AF700 (eBioscience, 30-F11, 56045182), MHCII-APC-eFluor780 (eBioscience, M5/114152, 47532182), Siglec-F-PE (BD Pharmingen, E502440, 552126), B220-PE-Cy5 (BD Pharmingen, RA36B2, 553091), CD317-PECy7<br>(eBioscience, eBio927, 25317282), CD3-BUV395 (BD Horizon, 1452C11, 563565), TCRb chain PE/Cy7 (Biolegend, H57597,

	109222), FoxP3-APC (eBioscience, 17577382), TCRgd-AF488 (Biolegend, GL3; 118128); and Fc receptor-blocking antibody CD16/CD32 (clone 2.4G2, BD Biosciences). For immunofluorescense microscopy, dewaxed paraffin or frozen skin sections or horizontal tail wholemounts were labelled with anti-filaggrin Ab (1:1000, Covance, PRB-417P), anti-F4/80 Ab (1:1000, AbD Serotec, MCA497G), CD11b-PE (1:300; eBioscience, M1/70, 12-0112-81), CD45 (1:500; BD Pharmingen, 30-F11, 550539), Keratin-6A Ab (1:1000, Biolegend), Keratin-14 (1:1000, Abcam, LL002, ab7800), Ki-67 Ab (1: 1000; Cell Signaling Technology, D3B5, 12202) or cleaved caspase-3 Ab (1: 1000; Cell Signaling Technology, 9661). As secondary antibodies donkey-anti-mouse 488 AlexaFluor (1:2000) and goat-anti-rabbit DyLight 586 (1:2000) were used in combination with Dapi.
Validation	Antibodies were validated for use in mouse skin by flow cytometry or immunofluoresence.

Animals and other organisms

Policy information about studies involving animals; ARRIVE guidelines recommended for reporting animal research

Laboratory animals	The following mouse lines were used: OTULIN floxed (Verboom et al., 2020), K14-Cre (Hafner et al., 2004), Fadd floxed (Mc Guire et al., 2010), Mlkl floxed (Murphy et al., 2013), Ripk1D138N [53], casp8-/- (Salmena L et al., 2003), Ripk3-/- (Newton et al., 2004), Tnfr1 -/- (Pfeffer, K., et al., 1993), Ifnar1-/- (Muller, U., et al., 1994), Myd88-/- (Adachi et al.., 1998) and IL1R-/- (Labow, M., et al., 1997). All alleles were maintained on a C57BL/6 genetic background. Both female and male mice were used at the age indicated in the relevant figure legends. Mice were used at different ages, as indicated in the appropriate figure legends.
Wild animals	The study did not involve wild animals
Field-collected samples	The study did not involve samples collected from fields
Ethics oversight	All experiments on mice were conducted according to institutional, national and European animal regulations for animal testing and research. Animal protocols were approved by the VIB-Ghent University ethical review board.

Note that full information on the approval of the study protocol must also be provided in the manuscript.

Flow Cytometry

Plots

Confirm that:

- [x] The axis labels state the marker and fluorochrome used (e.g. CD4-FITC).
- [x] The axis scales are clearly visible. Include numbers along axes only for bottom left plot of group (a 'group' is an analysis of identical markers).
- [x] All plots are contour plots with outliers or pseudocolor plots.
- [x] A numerical value for number of cells or percentage (with statistics) is provided.

Methodology

Sample preparation	Flow cytometric analysis of total mouse back skin (from 8-weeks-old mice) was performed on single-cell suspensions obtained following trypsin digestion for 1 hour at 37°C and subsequent digestion with collagenase type-1 (1,25 mg/ml), type-2 (0,5 mg/ml) and type-4 (0,5 mg/ml) for 30 to 45 min.
Instrument	Measurements were performed on a BD Fortessa 5-laser cytometer
Software	Data were analysed using FlowJo 10.6.1. software.
Cell population abundance	Prior to measuring, counting beads (Life Technologies) were added to the cells.
Gating strategy	Gating strategy for different immune cell populations is exemplified in Supplementary Figure 4b.

- [x] Tick this box to confirm that a figure exemplifying the gating strategy is provided in the Supplementary Information.
